# Differentiated PDGFRα-Positive Cells: A Novel In-Vitro Model for Functional Studies of Neuronal Nitric Oxide Synthase

**DOI:** 10.3390/ijms22073514

**Published:** 2021-03-29

**Authors:** Bashair M. Mussa, Amir Ali Khan, Ankita Srivastava, Sallam Hasan Abdallah

**Affiliations:** 1 Basic Medical Sciences Department, College of Medicine, University of Sharjah, Sharjah 27272, United Arab Emirates; 2 Department of Applied Biology, College of Science, University of Sharjah, Sharjah 27272, United Arab Emirates; amkhan@sharjah.ac.ae; 3 Sharjah Institute for Medical Research, University of Sharjah, Sharjah 27272, United Arab Emirates; ankita2112@gmail.com; 4 Research Institute of Sciences & Engineering, University of Sharjah, Sharjah 27272, United Arab Emirates; sallam.abdallah@hotmail.com

**Keywords:** gastrointestinal motility disorders, diabetic gastroparesis, PDGFRα-positive cells, nitric oxide, nitric oxide synthase, hyperglycemia, mesenchymal stromal cells

## Abstract

It is evident that depletion of interstitial cells and dysfunction of nitric oxide (NO) pathways are key players in development of several gastrointestinal (GI) motility disorders such as diabetic gastroparesis (DGP). One of the main limitations of DGP research is the lack of isolation methods that are specific to interstitial cells, and therefore conducting functional studies is not feasible. The present study aims (i) to differentiate telomerase transformed mesenchymal stromal cells (iMSCs) into platelet-derived growth factor receptor-α-positive cells (PDGFRα-positive cells) using connective tissue growth factor (CTGF) and L-ascorbic acids; (ii) to investigate the effects of NO donor and inhibitor on the survival rate of differentiated PDGFRα-positive cells; and (iii) to evaluate the impact of increased glucose concentrations, mimicking diabetic hyperglycemia, on the gene expression of neuronal nitric oxide synthase (nNOS). A fibroblastic differentiation-induction medium supplemented with connective tissue growth factor was used to differentiate iMSCs into PDGFRα-positive cells. The medium was changed every day for 21 days to maintain the biological activity of the growth factors. Gene and protein expression, scanning electron and confocal microscopy, and flow cytometry analysis of several markers were conducted to confirm the differentiation process. Methyl tetrazolium cell viability, nitrite measurement assays, and immunostaining were used to investigate the effects of NO on PDGFRα-positive cells. The present study, for the first time, demonstrated the differentiation of iMSCs into PDGFRα-positive cells. The outcomes of the functional studies showed that SNAP (NO donor) increased the survival rate of differentiated PDGFRα-positive cells whereas LNNA (NO inhibitor) attenuated these effects. Further experimentations revealed that hyperglycemia produced a significant increase in expression of nNOS in PDGFRα-positive cells. Differentiation of iMSCs into PDGFRα-positive cells is a novel model to conduct functional studies and to investigate the involvement of NO pathways. This will help in identifying new therapeutic targets for treatment of DGP.

## 1. Introduction

Gastrointestinal (GI) motility disorders, such as diabetic gastroparesis (DGP), are characterized by a complex pathological nature, and several risk factors are yet to be identified [1]. Delayed gastric emptying is associated with drastic fluctuations in blood glucose and frequent episodes of hypoglycemia, representing a clinical challenge for patients to manage their diabetes. This leads to poor glycemic control and very limited therapeutic options for DGP [2,3,4,5]. Dysfunction of smooth muscle cells (SMCs) is a strong causative candidate of GI motility disorders and therefore, more efforts are exerted to investigate the role of dysfunctional SMCs in GI motility disorders. It is well documented that SMCs are complex tissues constituting various cell types, including myocytes, nerve cells, and several types of interstitial cells [6]. The latter have diverse origins; some have hematopoietic origins and are involved in innate immune responses and others have mesenchymal origins such as interstitial cells of Cajal (ICC), which are concerned with regulation of motility functions and neurotransmission and their depletion is a contributing factor to the pathogenesis of DGP [6,7,8].

In addition, fibroblast-like cells are recognized as a new class of interstitial cells; the name of these new cells has been substituted by more recent names: (i) Telocyte cells according to the ultrastructural morphology and (ii) platelet-derived growth factor receptor-α-positive cells (PDGFR-α-positive cells) according to the expression of PDGFR alpha marker [9]. Although there is a debate about which name should be used, the expression of the PDGFR alpha marker seemed to be more evident [9]. Therefore, in this study, PDGFR-α-positive cells will be used to refer to this new class of interstitial cells.

Although PDGFRα-positive cells and ICCs share the same mesenchymal origin, PDGFRα-positive cells have distinguishable microscopic features including moderate to high electron density and mature rough endoplasmic reticulum [10]. Mirroring the distribution of ICCs, PDGFRα-positive cells are widely distributed in the GI smooth muscles suggesting an involvement of these cells in the regulation of GI motility [10,11,12]. In addition, their approximation as well as the gap junctions that they share with SMCs, suggests that these cells play a crucial role in signal neurotransmission to influence muscle tone [13,14]. However, there is huge amount of speculation about their exact role and involvement in GI motility disorders. This is mainly owing to the lack of isolation methods that are specific to this cell-type and the fact that conducting functional studies using these cells is considered as a challenge [13].

It is noteworthy that some studies have suggested the presence of these cells in other systems such as the urinary tract [15]. However, more recent studies have demonstrated that PDGFRα-positive cells selectively expressed the SK3 channel among all the interstitial cells that are present in the gut interstitium [16]. The dysfunction of SK3 channels is associated with several GI disorders including DGP, and this highlights the possible involvement of PDGFRα-positive cells in neurotransmission [17]. More importantly, it is believed that these cells play a key role as neural transducers, responding, in particular, to nitric oxide (NO) as they contain soluble guanylyl cyclase [18]. Interestingly, PDGFRα-positive cells express nitric oxide-sensitive guanylyl cyclase (NO-GC), indicating that PDGFRα-positive cells might be the mediator of NO action on GI smooth muscles, which is found to be diminished in DGP [14,19].

NO is a neuronally derived inhibitory neurotransmitter that plays a prominent role in several GI functions including GI secretion, smooth muscle relaxation, and motility [20,21]. The effect of NO is mainly mediated through NO-GC and further transduced by cGMP-dependent mechanisms [18]. Although there are three isoforms of nitric oxide synthase (NOS): neuronal (nNOS), endothelial (eNOS), and inducible (iNOS), emerging evidence has shown that impairment in the expression of nNOS contributes to the pathogenesis of DGP [21,22]. Interestingly, it has been found that mutation in the nNOS gene leads to diabetic gastropathy and that the expression of nNOS can be restored by insulin treatment [20,22,23].

Several studies have emphasized the role of NO on the viability of ICCs, but there is no study that focuses on the effect of NO on PDGFRα-positive cells [24]. Given the close association between ICCs and PDGFRα-positive cells, we propose that NO is a survival factor for PDGFRα-positive cells and that the effect of NO on smooth muscle relaxation and motility might be mediated through PDGFRα-positive cells.

Furthermore, recent research conducted by Tong et al. encourages the idea of obtaining PDGFRα-positive cells via controlled differentiation of MSCs in-vitro [25]. Using an appropriate combination of growth factors and nutritional media, it is possible to generate PDGFRα-positive cells [25,26]. Based on these findings, our proposal is to establish an in-vitro model using PDGFRα-positive cells for studying the involvement of NO in normoglycemic and hyperglycemic conditions. Moreover, the ability to have a non-invasive approach towards studying SMCs will help to understand the role of PDGFRα-positive cells in various clinical conditions including DGP. This will also highlight their importance as potential and futuristic biomarkers for diagnosis of DGP.

Accordingly, the present study aims to (i) differentiate telomerase transformed mesenchymal stromal cells (iMSCs) into PDGFRα-positive cells using connective tissue growth factor (CTGF) and L-ascorbic acids (LAA); (ii) investigate the effects of NO donor and inhibitor on the survival rate of differentiated PDGFRα-positive cells, and (iii) evaluate the impact of increased glucose concentrations mimicking diabetic hyperglycemia on the gene expression of nNOS.

## 2. Results

### 2.1. Differentiation of iMSCs into PDGFRα-Positive Cells

**Figure 1:**
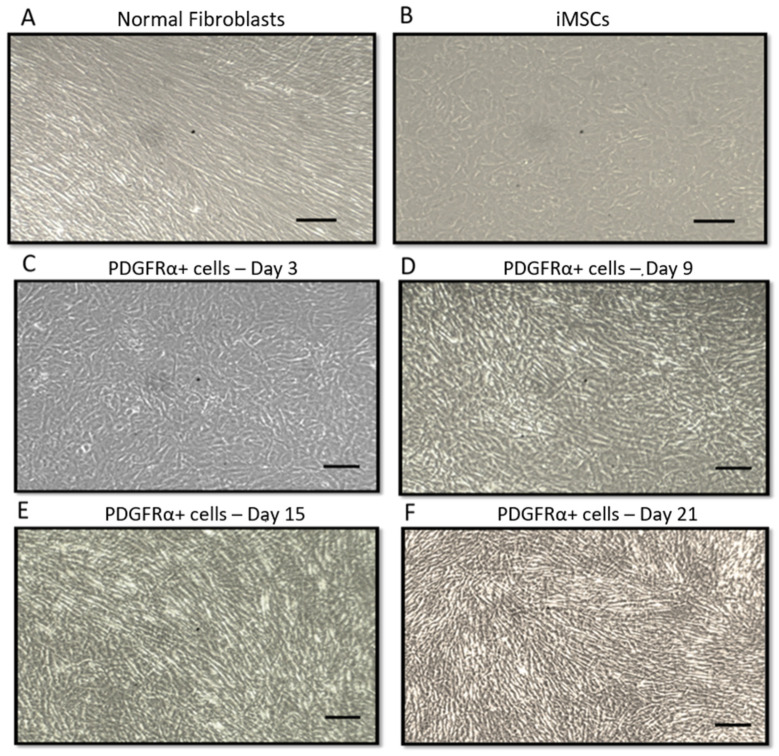
Morphological changes associated with differentiation of immortalized human bone marrow derived mesenchymal stromal cells (iMSCs) into platelet-derived growth factor receptor-α-positive cells (PDGFRα-positive cell) using contrast microscopy. Representative microscope images showing the morphological features of (**A**). Normal fibroblasts. (**B**). Morphological features of iMSCs cells prior to the differentiation process. (**C**–**F**). Differentiation of iMSCs into PDGFRα-positive cells as shown by Day 3, Day 9, Day 15, and Day 21, respectively (*n* = 3, Scale bar = 10 nm).

**Figure 2:**
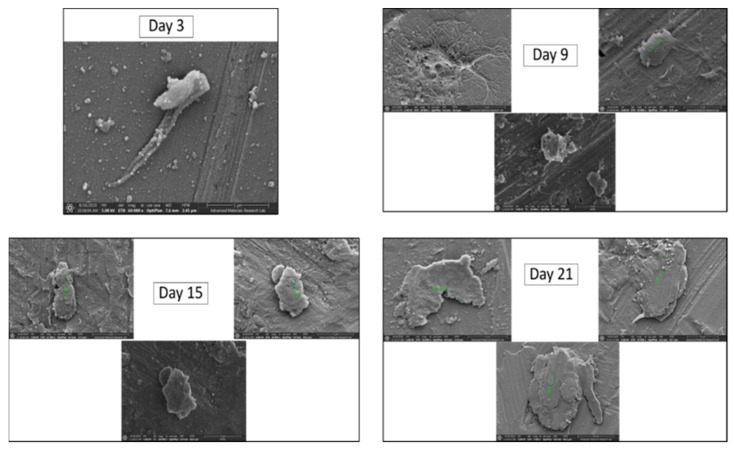
SEM images showing the morphological changes in iMSCs as it progresses towards differentiated PDGFRα-positive cells. The samples were fixed and dehydrated on day 3, 9, 15, and 21 of the experiment.

In the first group of experiments, we differentiated iMSCs into PDGFRα-positive cells. Exposure of iMSCs to fibroblastic differentiation-induction medium led to morphological changes that were monitored for 21 days. Microscope images were taken every two days and the process of differentiation was observed closely. As shown in Figure 1, PDGFRα-positive cells exhibited noticeable morphological changes including elongation and exhibition of spindle shaped fibers (Figure 1F), which were distinct from normal fibroblasts (Figure 1A) and normal iMSCs (Figure 1B). As shown in Figure 1C–F, the changes throughout the 21 days clearly indicated that iMSCs were differentiated into PDGFRα-positive cells. In addition, the morphological changes during the differentiation process of iMSCs into PDGFRα-positive cells were assessed using scanning electron microscope (SEM), and images were captured at day 3, 9, 15, and 21 of the experiment and showed significant changes in the structure and size of the cells (Figure 2). The increase in the diameter of the cells was directly proportional to the days of the differentiation, from ~2 µm in day 9 to ~30 µm in day 21. This was supported by previous reports that used SEM to further confirm the differentiation of MSCs [27,28].

### 2.2. Differential Gene Expression of Extracellular Matrix Proteins in Fibroblasts, iMSCs and PDGFRα-Positive Cells

**Figure 3:**
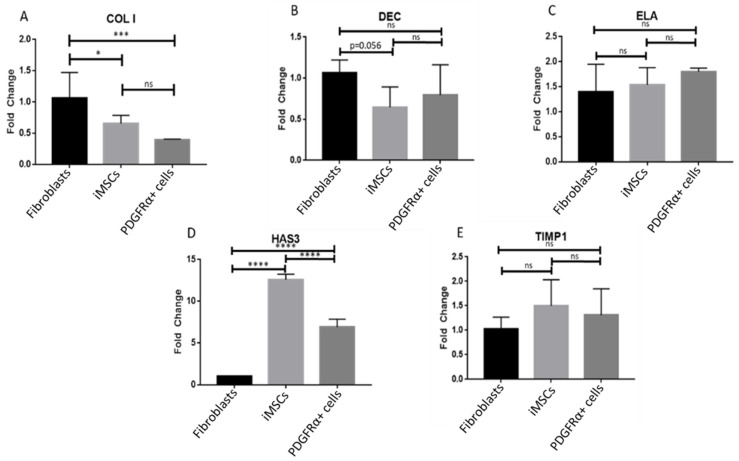
Gene expression of ECM proteins in fibroblasts, iMSCs, and PDGFRα-positive cells. qPCR analysis performed after 21 days of culture showed differential expression of extracellular matrix (ECM) proteins. (**A**–**E**). show gene expression of COL I, DEC, ELA, HAS3 and TIMP1, respectively. Gene expression of COL I was significantly different in fibroblasts compared to both iMSCs and PDGFRα-positive cells individually. Gene expression of HAS3 was significantly different between fibroblasts, iMSCs, and PDGFRα-positive cells (*n* = 3, * *p* < 0.05, *** *p* < 0.001, **** *p* < 0.0001); ns: not significant.

Quantitative PCR analysis showed differential gene expression of extracellular matrix (ECM) proteins in fibroblasts, iMSCs, and PDGFRα-positive cells. Five ECM proteins (COL I, DEC, ELA, HAS3, and TIMP1) were tested and statistical significance was used to mark the difference between their relative gene expression. Gene expression of COL I was significantly higher in fibroblasts as compared to both iMSCs and the derived PDGFRα-positive cells (Figure 3A). A pronounced increase was observed in the expression of HAS3 in iMSCs and PDGFRα-positive cells whereas fibroblasts showed very low expression (Figure 3D). Furthermore, the expression of HAS3 in PDGFRα-positive cells was higher than in fibroblasts but much lower than iMSCs (Figure 3D). Increased expression of HAS3 is one of the main differentiation features of PDGFRα-positive cells. Gene expression of DEC, ELA, and TIMP1 exhibited variations amongst fibroblasts, iMSCs, and PDGFRα-positive cells. However, none of them were statistically significant (Figure 3B,C,E). Further investigation of the differentiation process included gene and protein expression of markers that were used to confirm this process, previously [25]. Differential gene and protein expression of these markers are described in the following subsections.

### 2.3. Gene and Protein Expression of Stem Cell Differentiation Markers in Fibroblasts, iMSCs, and PDGFRα-Positive Cells

**Figure 4:**
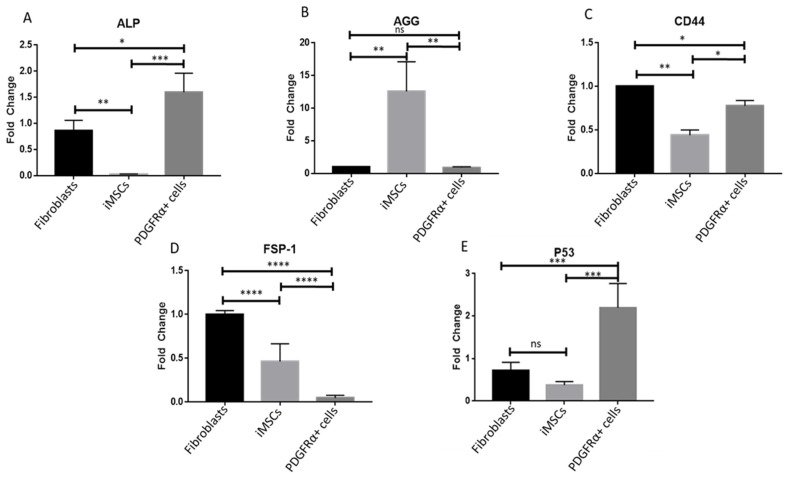
Gene expression of stem cell differentiation (SCD) markers in fibroblasts, iMSCs and PDGFRα-positive cells. qPCR analysis performed after 21 days of culture showed differential expression of SCD markers. (**A**–**E**). show gene expression of ALP, AGG, CD44, FSP-1 and P53. Gene expressions of ALP, CD44, and FSP-1 were significantly different between fibroblasts, iMSCs, and PDGFRα-positive cells (*n* = 3, * *p* < 0.05, ** *p* < 0.01, *** *p* < 0.001, **** *p* < 0.0001); ns: not significant.

Investigation of the gene expression of stem cell differentiation (SCD) markers showed that mRNA levels of ALP were significantly higher in PDGFRα-positive cells compared to iMSCs and fibroblasts (Figure 4A). Expression of ALP in fibroblasts was about 50% less compared to PDGFRα-positive cells and 100% more compared to iMSCs which showed almost no ALP expression (Figure 4A). Increased expression of ALP in the differentiated cells was shown in previous reports (25). As shown in Figure 4B, gene expression of AGG was significantly higher in iMSCs compared to fibroblasts and PDGFRα-positive cells. Comparison of mRNA levels of CD44 and FSP-1 showed that both of these genes were highly expressed in fibroblasts whereas their expression in iMSCs and PDGFRα-positive cells was inconsistent (Figure 4C,D). Although CD44 expression was much less in PDGFRα-positive cells, a more significant increase in expression of CD44 was observed in these cells compared to iMSCs (Figure 4C). However, expression of FSP-1 in iMSCs was lower than in fibroblasts and significantly higher in PDGFRα-positive cells (Figure 4D). Assessment of pro-apoptotic gene p53 in fibroblasts, iMSCs, and PDGFRα-positive cells showed that the mRNA levels of p53 were significantly higher in PDGFRα-positive cells compared to fibroblasts and iMSCs (Figure 4E).

**Figure 5:**
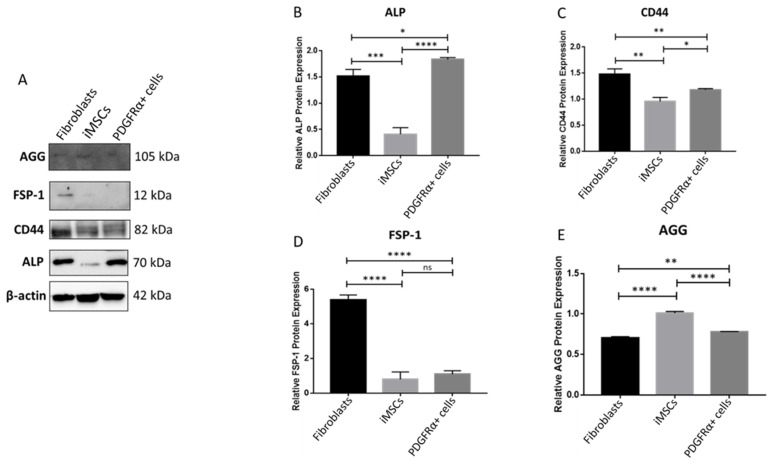
Protein expression of SCD markers in fibroblasts, iMSCs, and differentiated PDGFRα-positive cells. (**A**). Western blot images for ALP (70 kDa), CD44 (82 kDa), FSP-1 (12 kDa), and AGG (105 kDa) along with the loading control β-actin (42 kDa) observed on the same blot. (**B**–**E**). show the corresponding histograms of SCD marker’s protein expression calculated relative to β-actin. (*n* = 2, * *p* < 0.05, ** *p* < 0.01, *** *p* < 0.001, **** *p* < 0.0001); ns: not significant.

Assessment of protein expression of the SCD markers in fibroblasts, iMSCs, and differentiated PDGFRα-positive cells showed comparable findings to the gene expression (Figure 5A–D). Figure 5A shows western blot images for ALP (70 kDa), CD44 (82 kDa), FSP-1 (12 kDa), and AGG (105 kDa) along with the loading control β-actin (42 kDa). Protein expression of ALP was significantly higher in PDGFRα-positive cells compared to fibroblasts and iMSCs. Protein expression of the ALP in the latter was less than 20% compared to the expression in PDGFRα-positive cells (Figure 5B). Protein levels of CD44 were high in fibroblasts whereas their expression in iMSCs and PDGFRα-positive cells was significantly less (Figure 5B). Although CD44 protein expression was much less in PDGFRα-positive cells, a more significant increase in expression of CD44 was observed in PDGFRα-positive cells compared to iMSCs (Figure 5C). Protein expression of FSP-1 was significantly high in fibroblasts compared to in iMSCs and PDGFRα-positive cells (Figure 5D). In addition, protein expression of AGG was significantly higher in iMSCs compared to fibroblasts and PDGFRα-positive cells (Figure 5E).

### 2.4. Assessment of Gastrointestinal Surface Markers in Fibroblasts, iMSCs, and PDGFRα-Positive Cells

**Figure 6:**
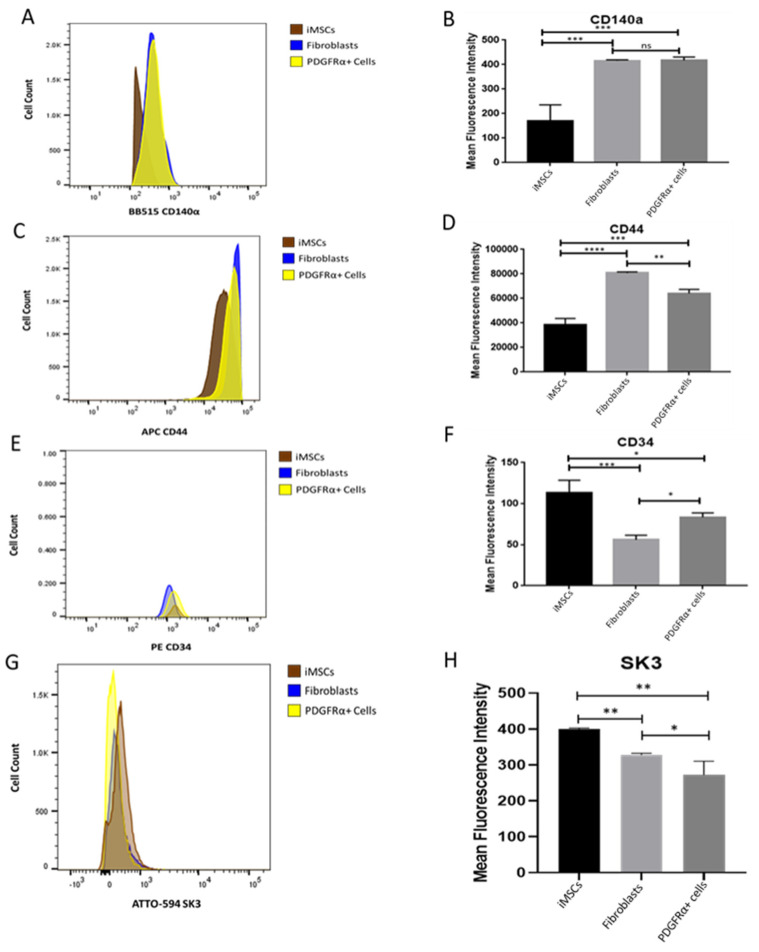
Expression of CD140α, CD44, CD34 and SK3 cell surface markers in iMSCs, fibroblasts, and PDGFRα-positive cells. Flow cytometry analysis detected the expression of CD140α ((**A**). cell count; (**B)**. mean fluorescence intensity), CD44 ((**C**). cell count; (**D**). mean fluorescence intensity), CD34 ((**E**). cell count; (**F**). mean fluorescence intensity), and SK3 ((**G**). cell count; (**H**). mean fluorescence intensity) in iMSCs, fibroblasts, and PDGFRα-positive (*n* = 3, * *p* < 0.05, ** *p* < 0.01, *** *p* < 0.001, **** *p* < 0.0001); ns: not significant.

Further examination included the expression of GI surface markers: CD140α, CD44, CD34, and SK3 in fibroblasts, iMSCs, and PDGFRα-positive cells. These surface markers were utilized to characterize and distinguish PDGFRα-positive cells from the other cells. The outcomes of flow cytometry have shown that PDGFRα-positive cells shared similar CD140α profile with fibroblasts as indicated by the similarity in their cell count and mean fluorescence intensity (Figure 6A,B). This also indicates that iMSCs were efficiently differentiated into PDGFRα-positive cells. iMSCs exhibited different CD140α profile with less cell count and lower fluorescence intensity and this finding was statistically significant. Similarly, CD44 profile was comparable to CD140α as the cell count and mean fluorescence intensities were significantly higher in fibroblasts and PDGFRα-positive cells as compared to iMSCs. However, using the same parameters, PDGFRα-positive cells exhibited a significantly lower CD44 profile compared to fibroblasts (Figure 6C,D). Different results were reported regarding the profile of CD34 wherein iMSCs had significantly different CD34 profile compared to fibroblasts and PDGFRα-positive cells, which was characterized by lower cell count as well as mean fluorescence intensity. CD34 profile of PDGFRα-positive cells was distinguished by significantly higher cell count and fluorescence intensity compared to fibroblasts. Moreover, SK3 profile showed distinguishable characteristics amongst the three cells: Fibroblasts, iMSCs, and PDGFRα-positive cells (Figure 6G,H).

### 2.5. Effects of Nitric Oxide on the Survival Rate of PDGFRα-Positive Cells

**Figure 7:**
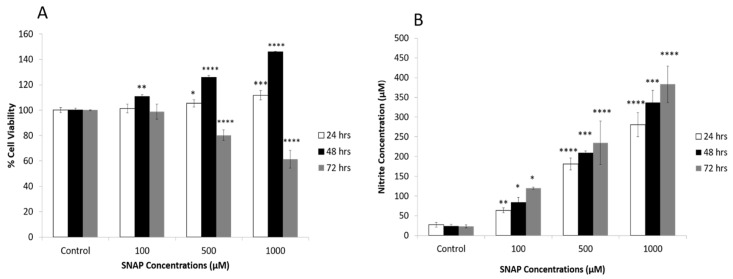
Effects of nitric oxide (NO) on the survival of the PDGFRα-positive cells. (**A**). NO donor, SNAP, increased the survival rate of the differentiated PDGFRα-positive cells significantly as compared to the control condition. This response was concentration- and duration-dependent for 24 and 48 h. Reduction in the survival rate of PDGFRα-positive cells was observed in response to treatment with SNAP for a longer duration (72 h). (**B**). Treatment of PDGFRα-positive with SNAP produced a significant increase in the level of nitrite in a concentration- and duration-dependent manner (*n* = 3, * *p* < 0.05, ** *p* < 0.01, *** *p* < 0.001, **** *p* < 0.0001).

Investigating the effects of NO on the survival rate of PDGFRα-positive cells showed that treatment with SNAP (NO donor; 100, 500 and 1000 µM) for 24 and 48 h increased the survival rate of the differentiated PDGFRα-positive cells significantly as compared to the control condition. As shown in Figure 7A, this response was both concentration- and duration-dependent. However, exposure of PDGFRα-positive cells to SNAP for a longer duration (72 h) produced an adverse effect on the survival rate of PDGFRα-positive cells. The measurement of nitrite, which is a primary stable and non-volatile breakdown product of NO, showed a concentration- and duration-dependent increase in nitrite levels in the PDGFRα-positive cells that were treated with SNAP (Figure 7B). As shown in the latter, nitrite concentrations were low (25 µM) in the control conditions and increased significantly in response to SNAP treatment.

### 2.6. Effects of Nitric Oxide Inhibition on the Survival Rate of PDGFRα-Positive Cells

**Figure 8:**
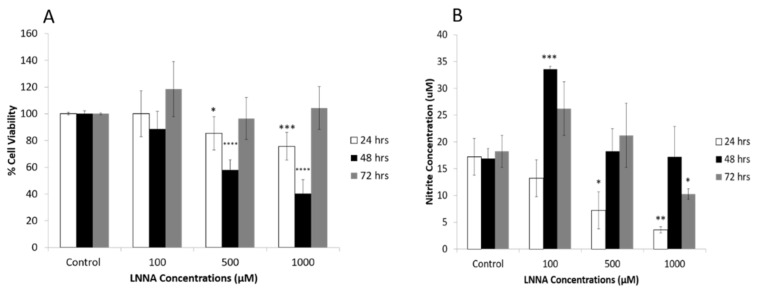
Effects of NO inhibitor on the survival of PDGFRα-positive cells. (**A**). Survival rate of the PDGFRα-positive cells decreased significantly in the presence of NO inhibitor, LNNA, and this response was concentration- and duration-dependent for 24 and 48 h. LNNA produced no change in the survival rate of PDGFRα-positive cells during the longer duration of 72 h. (**B**). Treatment of PDGFRα-positive cells with LNNA for 24 and 72 h produced a significant decrease in the level of nitrite and this response was concentration dependent. An increase and no change in the survival rate of PDGFRα-positive was observed during the treatment of 48 h (*n* = 3, * *p* < 0.05, ** *p* < 0.01, *** *p* < 0.001, **** *p* < 0.0001).

As shown in Figure 8A, treatment of PDGFRα-positive cells with LNNA (NO inhibitor; 100, 500, and 1000 µM), attenuated the survival rate of the PDGFRα-positive cells in a concentration- and duration-dependent fashion for 24 and 48 h as compared to the control condition. No change in the survival rate of PDGFRα-positive cells was observed after treatment of PDGFRα-positive cells with LNNA for a longer duration (72 h). A significant reduction in nitrite levels in PDGFRα-positive cells was observed after treatment with LNNA for 24 h as compared to the control condition. However, the treatment with LNNA (100 µM) for 48 h produced inconsistent responses as there was an increase in nitrite levels compared to the control conditions (Figure 8B). This response was about 50% less compared to the increased levels of nitrite in response to SNAP (100 µM–48 h) (Figure 6B).

### 2.7. Effects of Glucose on Gene Expression of Neuronal Nitric Oxide Synthase in PDGFRα-Positive Cells

**Figure 9:**
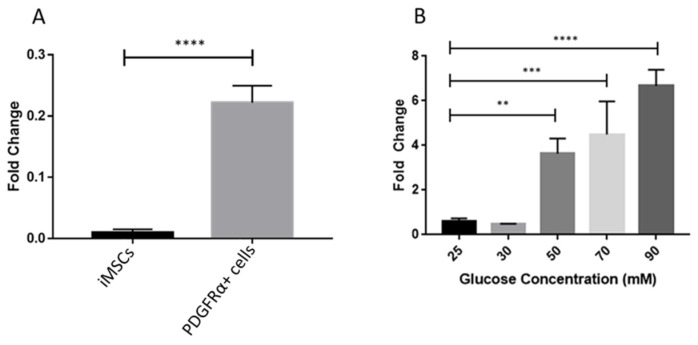
Effects of different glucose concentrations on gene expression of neuronal nitric oxide synthase (nNOS) in PDGFRα-positive cells. (**A**). Under normal concentration of glucose (25 mM), gene expression of nNOS was significantly higher in PDGFRα-positive cells compared to iMSCs. (**B**). Treatment of PDGFRα-positive cells with different concentrations (30, 50, 70, and 90 mM) of glucose produced a significant and concentration-dependent increase in gene expression of nNOS. (*n* = 3, ** *p* < 0.01, *** *p* < 0.001, **** *p* < 0.0001).

**Figure 10:**
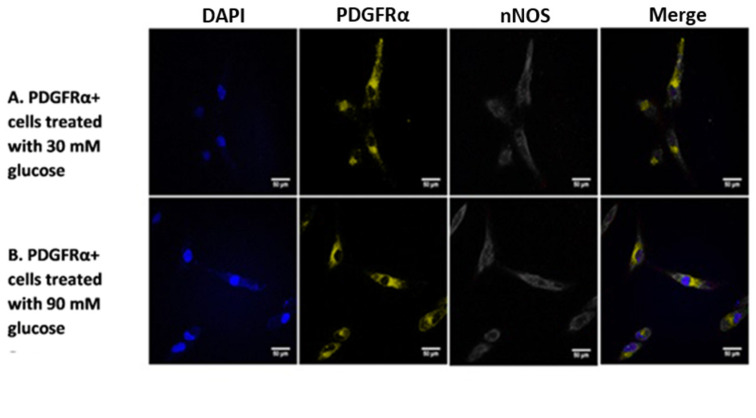

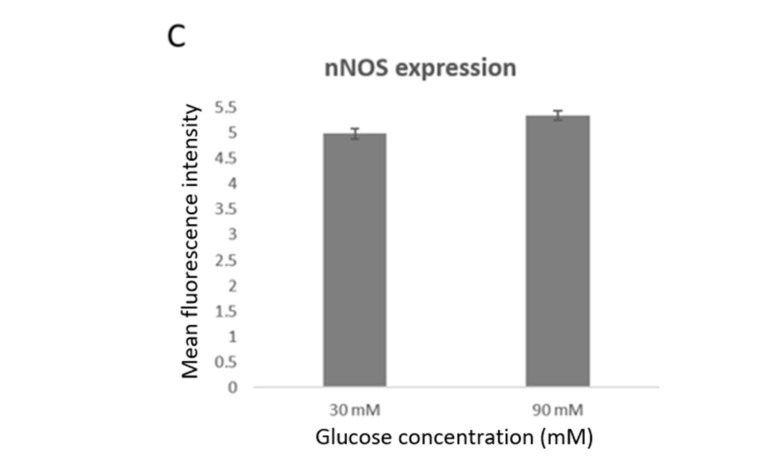
The expression of nNOS (gray) and PDGFRα (yellow) cell surface markers in differentiated PDGFRα-positive cells with nuclei counterstained with DAPI (blue). (**A**). Cells treated with 30 mM glucose. (**B**). Cells treated with 90 mM glucose. Merged images show expression of nNOS at 90 mM compared to 30 mM glucose concentration and due to the low number of cells that were used in imaging studies, the difference in expression of nNOS at 90 mM and 30 mM was not as clear as in the gene and protein expression. (**C**). The mean of fluorescence intensity of the signals for the stained cells that was extracted from the software image J after analysis of the pictures. Scale bar = 50 μm.

**Figure 11:**
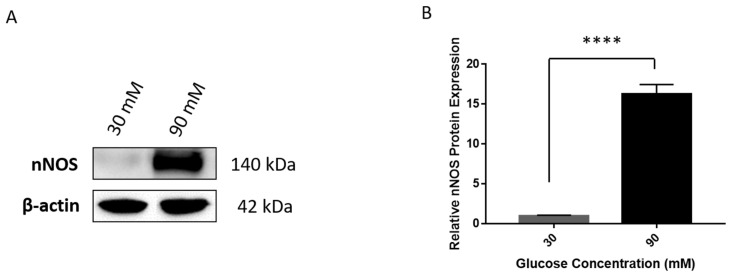
Protein expression of nNOS at 30 mM and 90 mM glucose concentrations in differentiated PDGFRα-positive cells. (**A**). Western blot images for nNOS observed at 140 kDa and the loading control β-actin at 42 kDa on the same blot. (**B**). The corresponding histogram of nNOS protein expression calculated relative to β-actin with a highly significant expression observed at 90 mM (**** *p* < 0.0001; *n* = 2) as compared to 30 mM glucose treatment.

Investigating the presence of nNOS showed that its expression was significantly higher in PDGFRα-positive cells as compared to iMSCs under normal glucose concentration of 25 mM (Figure 9A). Further experiments assessed the effects of increased levels of glucose on nNOS gene expression in PDGFRα-positive cells. Glucose concentrations of 25 mM and 30 mM had no significant effect on the gene expression of nNOS. However, treating PDGFRα-positive cells with higher doses of glucose led to a significant, concentration-dependent increase in the expression of nNOS (Figure 9B). In addition, we conducted immunostaining to examine the expression of nNOS in PDGFRa-positive cells at two concentration of glucose 30 mM and 90 mM (Figure 10A,B, respectively). Figure 10C shows the mean of fluorescence intensity of the signals for the stained cells that was extracted from the software image J after analysis of the pictures. Although the expression of nNOS seems to be a little higher under 90 nM glucose concentration, it is not statistically significant and the small number of cells that were used in the imaging studies could explain these results. Further experiments showed protein expression of nNOS at 30 mM and 90 mM glucose concentrations in differentiated PDGFRα-positive cells using western blotting, as shown in Figure 11. A significant increase in protein expression of nNOS was observed at a glucose concentration of 90 mM compared to 30 mM (Figure 11A,B).

## 3. Discussion

The mesenchymal origin and regulatory functions of interstitial cells has been suggested by previous reports [6]. However, fibroblastic differentiation of MSCs became feasible by using a differentiation-inducing medium incorporated with CTGF and LAA [25]. In the present study, different methods were used to confirm that iMSCs were differentiated into PDGFRα-positive cells. These methods include SEM, gene and protein expression, flow cytometry, and immunostaining, and the outcomes of these experimentations have shown clearly that PDGFRα-positive cells were distinguishable from iMSCs. PDGFRα-positive cells were used as a novel model to investigate the effects of NO on their survival and to study the expression of NOS under normo- and hyper-glycemic conditions [26].

The main difference between iMSCs and fibroblasts is that the former are stem cells and can form colony-forming units while the latter express ECM proteins in the tissue [29]. The iMSCs have broader differentiation potential despite the morphological similarity of these two cell types [29,30].

Measuring the expression of ECM proteins has shown that the two main fibrous proteins, COL I and ELA, were expressed differentially in the fibroblasts, iMSCs, and PDGFRα-positive cells. Unexpectedly, gene expression of COL I was low in PDGFRα-positive cells and iMSCs as compared to fibroblasts. Although increased levels of COL I in the PDGFRα-positive cells were demonstrated previously, in contrast, there are other reports where it was found that the COL I mRNA level was lowered upon addition of CTGF [25,31]. Further findings showed that CTGF regulates ECM proteins more than their corresponding mRNAs [25]. In our study, COL I mRNA was also lowered in the presence of CTGF. In previous studies, ECM protein levels were shown to consistently increase in response to CTGF [32]. In agreement with previous studies, no significant changes were observed in gene expression of ELA in presence of CTGF and the levels were comparable to fibroblasts [25]. The origin of fibroblasts seems to influence the gene expression of ELA. It was found that fibroblasts from upper dermal layer exhibited higher levels of ELA mRNA whereas lower dermal fibroblasts had the lowest levels of ELA mRNA indicating that fibroblasts vary depending on their origin [33]. DEC is one of the most important ECM proteins involved in organization of collagen fibers [34]. The present results have shown a trend toward an increase in gene expression of DEC upon addition of CTGF to further confirm the differentiation process. In line with this finding, a study by Vial et al. has demonstrated that CTGF enhanced the synthesis of DEC, which, in turn, interacts with CTGF to regulate its biological activity [35]. Levels of HAS3 mRNA were found to be high in iMSCs and this was attributed to the ability of these cells to secrete high levels of HAS3 [36]. However, these levels seemed to be lower in PDGFRα-positive cells and yet higher compared to fibroblasts. Tong et al. showed that HAS3 expression was upregulated in response to CTGF, but this was conducted in fibrous scaffolds based on poly (glycerol sebacate) and poly(e-caprolactone) (PGS-PCL) without any comparison with the normal fibroblasts [25]. Examination of TIMP1 expression revealed that CTGF did not affect the gene expression of glycoprotein in PDGFRα-positive cells and its level was comparable between fibroblasts, iMSCs, and PDGFRα-positive cells. TIMP is a family of four members: TIMP-1, TIMP-2, TIMP-3, and TIMP-4, which respond differently to CTGF [37]. This was supported by a study conducted on renal interstitial fibroblasts, which provided evidence that CTGF increased the gene expression of TIMP-2 and thus enhanced the ECM remodeling [37]. No previous studies have shown the involvement of TIMP1 in this process.

Interestingly, it was found that mRNA levels of ALP were significantly higher in PDGFRα-positive cells compared to fibroblasts and iMSCs and can be considered as a main marker of PDGFRα-positive cells. Given that ALP is a classic indicator of osteogenesis and previous studies have reported low gene expression in response to CTGF, the present findings suggest that the differentiated PDGFRα-positive cells have distinct phenotypic features compared to the PDGFRα-positive cells that have been identified previously [13]. Fibroblasts can also be differentiated into osteoblasts and the expression of ALP indicates this feature [38]. In addition, CD44 is one of the markers of MSCs and several previous studies have demonstrated a significant reduction in CD44 expression as MSCs differentiated to fibroblasts [13]. In this study, CD44 gene expression in iMSCs was much less than what was observed in other studies. In this study, we used genetically engineered iMSCs and the forced expression of the telomerase enzyme might have affected the expression of CD44. This lack of CD44 expression in iMSCs was confirmed by Qian et al. in a subset of MSCs [39]. Furthermore, the lack of strong expression of FSP-1, as a fibroblast marker of epithelial-mesenchymal transition in the PDGFRα-positive cells, raised a question about the origin of these cells. It seemed that there are different subpopulations of PDGFRα-positive cells based on their mesenchymal origin, therefore further experimentation was conducted to investigate the nature of the differentiated PDGFRα-positive cells in this study [40]. PDGFRα-positive cells have the highest expression of p53 amongst the three cell types. Either the media composition or differentiation process that continued for 21 days might increase its expression; however, further research is warranted to unravel the causes for this differential expression. One study indicates that the knockdown of p53 in fibroblasts enhanced their differentiation into neurons [41].

Expression of gastrointestinal PDGFRα-positive cells markers were also assessed in the present study. Expression of CD140α (also known as PDGFRα) in the MSCs was much less as compared to fibroblasts and PDGFRα-positive cells emphasizing the specificity of this surface marker to PDGFRα-positive cells. One can urge that fibroblasts also exhibited a significant expression of CD140α/PDGFRα. However, this is the first study to compare the expression of CD140α in fibroblasts and PDGFRα-positive cells and this surface marker is usually used to distinguish between ICCs and PDGFRα-positive cells. In addition, PDGFRα is considered as an emerging, novel marker that needs to be studied in more depth to deduce its functional profile [42]. Expression of CD34, a sialomucin cell adhesion protein, has been used previously to differentiate PDGFRα-positive cells from ICCs [13]. However, in the present study, both PDGFRα-positive cells and iMSCs showed a significant increase in CD34 expression. There is a debate in the literature over the expression of this marker in iMSCs as for a long time these cells were considered as CD34-negative. However, more recent studies have challenged this opinion by demonstrating the expression of CD34 in MSCs [43,44]. Although the three cells, fibroblasts, PDGFRα-positive cells, and iMSCs, show different levels of SK3 expression, the cell count of PDGFRα-positive cells was higher; however, fluorescent intensity of SK3 was high in iMSCs. These results were not expected as SK3 is one of the main makers of PDGFRα-positive cells; however, this could suggest that iMSCs may also express SK3. Interestingly, other reports have demonstrated that the expression of different subtypes of SK channel were detectable in embryonic stem cells [45]. In addition, more recent studies have suggested that calcium-activated potassium channels play a role in controlling the differentiation process of iMSCs [46]. These findings shaped our future directions to optimize our present protocol and enhance the differentiation process of PDGFRα-positive cells.

PDGFRα-positive cells are anatomically proximate to inhibitory motor neurons, which are believed to be nitrergic neurons, and it was also found that PDGFRα-positive cells express post-junctional receptors for NO [47]. This has addressed the possibility that PDGFRα-positive cells might be responsive to and interactive with nitrergic inputs [47]. These inputs seemed to be involved in the control of several GI functions including gastric emptying and GI smooth muscle relaxation and motility [5].

Interestingly, low levels of NO were detected in the control conditions suggesting that PDGFRα-positive cells release NO in a spontaneous manner. This is not a new concept as previous studies have demonstrated NO production from human dermal fibroblasts under normal physiological conditions [48]. In agreement with previous findings that demonstrated that NO is a survival factor for ICC, the data presented in this study have shown that the survival rate of PDGFRα-positive cells was enhanced in response to NO [22]. This was further supported by the finding that the survival of PDGFRα-positive cells was jeopardized when these cells were treated with NO inhibitor. It has been suggested that the protective function of NO is mediated via several mechanisms including removal of cellular lipid and protein radicals, and regulation of cell signaling processes at tight junction proteins [49].

After the differentiation process was completed, PDGFRα-positive cells were used in further experimentations to conduct functional studies to investigate the effects of NO on the survival rate of these cells and the impact of hyperglycemia in nNOS expression in PDGFRα-positive cells. It is noteworthy that the treatment of PDGFRα-positive cells with an NO donor for a long duration (72 h) failed to maintain the survival of PDGFRα-positive cells. A large amount of the literature has discussed the factors that influence the action of NO including type of cells, intracellular pathways, site of production, and duration of exposure [50,51,52,53]. It seems that the latter was the contributing factor to the reduction in the survival rate of PDGFRα-positive cells. NO can cause apoptotic or necrotic cell death via initiation of oxidative stress, dysregulation of cytosolic calcium, and DNA damage [50]. In addition, the adverse effects of NO inhibitor treatment on the survival rate of PDGFRα-positive cells were blocked after a long duration of exposure (72 h). This further supports the suggestion that the mechanistic profile of NO in PDGFRα-positive cells varies depending on the duration of exposure. Moreover, levels of nitrite were high compared to the control conditions when treated with NO inhibitor, however they were still about 50% less than the response to NO donor. Given that LNNA is a competitive inhibitor, it seems that more time and high concentrations are required to produced noticeable effects.

Previous studies have defined nNOS as the main source of NO, which acts as inhibitory neurotransmitter in GI, therefore the present study has investigated the expression of nNOS in iMSCs and PDGFRα-positive cells under normal glycemic condition [54]. Expression of nNOS was significantly higher in PDGFRα-positive cells compared to iMSCs emphasizing the fibroblastic differentiation from these cells and excluding the possibility that iMSCs express nNOS. It is noteworthy that the expression of different NOS isoforms has been identified in MSCs such as eNOS and iNOS. However, it is not evident that nNOS is expressed in iMSCs [55,56].

The present study has investigated the effects of hyperglycemia on the expression of nNOS in differentiated PDGFRα-positive cells. A significant increase in the expression of nNOS was observed in PDGFRα-positive cells in the presence of glucose and this response was concentration dependent. Although the present data showed that NO is important for the survival of PDGFRα-positive cells in normoglycemic conditions, the expression of nNOS in these cells was increased in hyperglycemic conditions. These findings address a critical question about the effects of NO on PDGFRα-positive cells: Are they beneficial or harmful? The main element in answering this question seems to be the concentration of glucose. This strongly supports the hypothesis that in normal conditions, NO supports the survival of the PDGFRα-positive cells; however, stressful events such as hyperglycemia can modulate the involvement of NO in cellular processes of the PDGFRα-positive cells. The dual function of NO is not a new concept; it has been demonstrated that certain concentrations of NO can react with superoxide anion to generate the peroxynitrite radical, which affects the function of different cells adversely [57]. In addition, a recent study by Adela et al. has shown that hyperglycemia increased the production of NO in patients with diabetes [58]. This further supports the importance of NO in diabetes, and to relate these findings to our results, future studies can include assessment of NO levels in patients with DGP.

The long differentiation protocol for 21 days influenced the survival rate of the differentiated cells adversely and led to inconsistency of expression of some markers. Future studies will focus on the optimization of the current protocol and expand the model to include three-dimensional cell culture.

In conclusion, the present study has demonstrated differentiation of iMSCs into PDGFRα-positive cells, which exhibited distinct properties. NO supported the survival of PDGFRα-positive cells under normoglycemic conditions, however nNOS expression increased significantly in response to hyperglycemia.

## 4. Materials and Methods

### 4.1. Cell Culture and Fibroblastic Differentiation

Telomerase-transformed immortalized human bone marrow derived mesenchymal stromal cells (iMSCs) (abm T0529; Richmond, Canada) were cultured in complete minimum essential alpha modification medium (MEM-α; Sigma Aldrich, St. Louis, MO, USA), supplemented with 0.292 g/L L-glutamine (Sigma Aldrich, St. Louis, MO, USA), 10% heat inactivated fetal bovine serum (FBS; Sigma Aldrich, St. Louis, MO, USA), and 1% penicillin-streptomycin (Sigma Aldrich, St. Louis, MO, USA) in a humidified atmosphere of 5% CO_2_ at 37 °C. When the cells reached 80% confluency, they were harvested using 0.25% trypsin-EDTA solution (Sigma Aldrich, St. Louis, MO, USA). iMSCs were then plated in 6-well plates (Jet Biofil, Guangzhou, China) at a density of 250 × 10^3^ cells/well. After the cells reached 70–80% confluency, they were exposed to a fibroblastic differentiation-induction medium that consisted of complete MEM-α (Sigma Aldrich, St. Louis, MO, USA) supplemented with 100 ng/mL CTGF (Biovendor, Brno, Czech Republic) and 50 µg/mL LAA (Sigma Aldrich, St. Louis, MO, USA) (25). The medium was changed every day for 21 days to maintain the biological activity of CTGF. The control group experiments were performed with iMSCs as negative and normal primary human fibroblasts (F180) as positive control in the absence of CTGF and LAA under the same culture conditions. On the 21st day, the differentiated PDGFRα-positive cells were harvested for further analysis.

To mimic hyperglycemic conditions, four concentrations of high glucose: 30 mM, 50 mM, 70 mM, and 90 mM, were prepared by adding 250 µL, 1250 µL, 2250 µL, and 3250 µL of 1M D-glucose (Sigma Aldrich, St. Louis, MO, USA), respectively, to 50 mL of MEM-α media containing initial normal glucose concentration of 25 mM (for each of the four concentrations).

### 4.2. Gene Expression

Upon completion of differentiation on day 21, PDGFRα-positive cells were harvested for RNA isolation using the PureLink RNA Mini Kit (Invitrogen, Carlsbad, CA, USA). Due to the high number of cells, sonication for 30–45 s using a rotor-stator cell homogenizer was used as the preferred method to disrupt the cell membrane. RNA was isolated following the manufacturer’s protocol with final elution volume of 50 µL. Isolated RNA was then quantified using the Nanodrop2000 spectrophotometer (Thermo Fisher Scientific, Waltham, MA, USA) and purity was determined by the A260/A280 ratio. From this, 0.5 µg RNA was used to reverse transcribe to cDNA in a final reaction volume of 20 µL containing 200 units of M-MLV reverse transcriptase in a reverse transcriptase buffer (Sigma Aldrich, St. Louis, MO, USA) along with 10 mM dNTP mix and 5 µM random primers from the High-Capacity cDNA synthesis kit (Applied Biosystems, Foster City, MA, USA). First, the reaction mix containing RNA, dNTPs, and random primers was incubated at 70 °C for 10 min. After this, M-MLV reverse transcriptase mix was added and the samples were incubated at 37 °C for 50 min followed by 90 °C for 10 min in a Veriti thermal cycler (Applied Biosystems, Foster City, MA, USA).

For analysis of gene expression, quantitative real-time PCR was performed using the Rotor Gene Q PCR system (Qiagen, Hilden, Germany) with GoTaq^®^ SYBR green master mix (Promega Corporation, Madison, WI, USA) in a total reaction volume of 20 µL. The cycling parameters included initialization at 95 °C for 2 min followed by denaturation at 95 °C for 15 s and annealing/extension at 60 °C for 1 min for a total of 40 cycles. To confirm the purity of the obtained PCR products, a melt curve involving sequential heating at 95 °C for 15 s, 60 °C for 1 min and 95 °C for 15 s was included. The primer sequences used for the specific amplification with collagen type I (COL I), decorin (DEC), elastin (ELA), hyaluronic acid synthase 3 (HAS3), tissue inhibitor of metalloproteinases 1 (TIMP1), CD44, fibroblast-specific protein (FSP-1), alkaline phosphatase (ALP), aggrecan (AGG), and apoptosis indicator p53 are listed in Table 1. For determining the fold change (relative gene expression) using the 2ΔΔCt method, glyceraldehyde-3-phosphate dehydrogenase (GAPDH) was used as the housekeeping gene. All primer sequences were obtained using their GenBank numbers as mentioned in the study by Tong et al. [25].

For gene expression analysis of nNOS, forward primer: 5′-TCCACCAGGAGATGCTCAACTAC-3′ and reverse primer 5′-TTCCAGACATGCGTGTTCCA-3′ were used.

### 4.3. Flow Cytometry Analysis

To confirm differentiation of PDGFRα-positive cells, four cell surface markers were selected: CD34-PE (BD Pharmingen, San Diego, CA, USA), CD44-APC (BD Pharmingen, San Diego, CA, USA), CD140α-BB515 (BD Horizon, Franklin Lakes, NJ, USA) and KCa2.3-ATTO-594 (SK3; Alomone Labs, Jerusalem, Israel). Upon completing differentiation on day 21, PDGFRα-positive cells were harvested using 0.5% trypsin-EDTA. Then, 0.5 × 10^6^ cells were counted using a hemocytometer (Marienfeld, Lauda-Königshofen, Germany) and centrifuged at 6000 RPM for 15 min at 4 °C to obtain a pellet. The pellet was washed twice with Dulbecco’s Phosphate Buffered Saline (PBS; Sigma-Aldrich, St. Louis, MO, USA) and re-suspended in 100 µL of FACS buffer containing 2% FBS and 1 mM EDTA (Sigma Aldrich, St. Louis, MO, USA). Recommended volumes of the specific cell surface marker antibodies as per their respective manufacturers were added to the cells and incubated at room temperature for 15–20 min before detection. A minimum of 15,000 gated events with forward and side light-scatter characteristics of viable cells were collected for analysis. Compensation was performed with human F180 fibroblasts as positive control and iMSCs as negative control. Flow cytometric analysis was conducted using the BD FACSAria™ III sorter (BD Biosciences, Franklin Lakes, NJ, USA).

### 4.4. Cell Viability

For measurement of cell viability, 1 × 10^4^ PDGFRα-positive cells were seeded per well in 96-well plates (Jet Biofil, Guangzhou, China) with 200 μL complete MEM-α culture medium and maintained in the incubator at 37 °C with 5% CO_2_. Upon reaching 80% confluency, cells were treated with 100 µM, 500 µM, and 1000 µM of S-nitroso-N-acetylpenicillamine (SNAP) and L-NG-nitro-L-arginine (LNNA). Cells without any treatment were regarded as controls. Cell viability was measured at 24-, 48-, and 72-h intervals using colorimetric assay with 3-[4,5-dimethylthiazol-2-yl]-2,5 diphenyltetrazolium bromide (MTT; Sigma-Aldrich, St. Louis, MO, USA). Culture medium was discarded and replaced with 20 μL of MTT (5 mg/mL) dissolved in 100 μL of PBS per well. Cells were then incubated at 37 °C for 2 h in the dark. Post incubation, this solution was discarded carefully and 100 μL of Dimethyl Sulfoxide (DMSO; Sigma-Aldrich, St. Louis, MO, USA) was added to each well to dissolve the formazan crystals formed from MTT salt reduction and the absorbance was recorded at 570 nm on a microplate reader. Percentage of cell viability was calculated from the average 570 nm absorbance value as per the following equation: % cell viability = (OD of sample at 570 nm/OD of control at 570 nm) × 100.

### 4.5. Nitrite Measurement Assay for Functional Analysis

To measure the concentration of nitrite, 50 µL of spent media was collected from each well of the same plate used for measurement of cell viability post treatment with SNAP and LNNA at 24-, 48-, and 72-h intervals. Nitrite concentration was then measured using the Greiss Reagent System (Promega Corporation, Madison, WI, USA) as per the manufacturer’s protocol. The absorbance was recorded at 595 nm in a microplate reader. To determine the concentration of nitrite, a standard reference curve was generated and used to compare the average absorbance value for each sample.

### 4.6. Protein Expression and Analysis

Approximately, 1 × 10^6^ PDGFRα-positive cells were collected at the end of differentiation on Day 21. Protein lysis was carried out using the M-PER™ mammalian protein extraction reagent (Thermo Fisher Scientific, Waltham, MA, USA) with the Halt™ Protease Inhibitor Cocktail (Thermo Fisher Scientific, Waltham, MA, USA) added to it in a 1:1000 dilution. Protein lysates were prepared as per the manufacturer’s instructions and quantified using the Bradford reagent (Biorad Laboratories, Hercules, CA, USA). 50 µg of protein was then loaded onto 10% SDS-PAGE gels for western blotting. The gels were transferred onto nitrocellulose membranes (Biorad Laboratories, Hercules, CA, USA) and incubated with blocking buffer (5% Skimmed Milk Powder in 1X Tris-buffered saline with 0.1% of Tween 20 [TBST]; Sigma Aldrich, St. Louis, MO, USA) for 1 h at room temperature following which primary antibodies for the following genes were added in the mentioned dilutions: anti-β actin (1:5000; Sigma Aldrich, St. Louis, MO, USA), anti-ALP (1:1000; ab83259), anti-CD44 (1:2000; ab189524), anti-S100A4 [FSP-1] (1:500; ab124805), and anti-Aggrecan [AGG] (1:100; ab3778) and incubated overnight at 4 °C. The next day, membranes were incubated for 1 h at room temperature with the appropriate secondary antibodies in a 1:1000 dilution (anti-mouse IgG [7076S] and anti-rabbit IgG [7074S], Cell Signaling Technology, Danvers, MA, USA) and visualized using the Clarity Western ECL substrate reagent (Biorad Laboratories, Hercules, CA, USA) in a Chemi-Doc Visualizer (Biorad Laboratories, Hercules, CA, USA). Simultaneously, protein expression was also determined in fibroblasts and iMSC (positive and negative control respectively) following the same protocol.

For determining the protein expression of nNOS, two concentrations, 30 mM and 90 mM glucose (lowest and highest), were chosen. At day 21 post differentiation, PDGFRα-positive cells were treated with the appropriate glucose concentrations and protein lysate was isolated using the above-mentioned protocol. Membranes were incubated with primary antibodies against β-actin and nNOS (1:1000; ab219373) and with secondary antibodies against mouse IgG and rabbit IgG, respectively, the following day.

All primary antibodies were purchased from Abcam, Cambridge, UK, unless mentioned otherwise. Bands were quantified using the ImageJ software.

### 4.7. Scanning Electron Microscopy

At pre-determined time points of day 3, 9, 15, and 21, PDGFRα-positive cells were first washed twice with PBS and then trypsinized with 0.05% 1X Trypsin-EDTA at room temperature for 1 min. Trypsin was discarded, and cells were incubated at 37 °C for 5 min. Detached cells were then resuspended in 3 mL of PBS and centrifuged at 1000 rpm for 10 min at 4 °C and this washing step was repeated twice. Washed cells were then fixed with 500 μL of fixative (2.5% glutaraldehyde in PBS buffer with 2% sucrose), vortexed, and kept on ice for 1 h. Additional 500 μL of PBS was added to the tube and centrifuged at 1000 rpm for 10 min at 4 °C. After centrifugation, supernatant was discarded and 20 μL of 1% osmium tetraoxide was added to the pellet and incubated on ice for 30 min. Additional 1 mL of PBS was added and centrifuged at 1000 rpm for 10 min at 4 °C to remove osmium tetraoxide. Graded dehydration was then carried out using 30% and 50% ethanol. Cells were finally fixed in 70% ethanol and stored at 4 °C until visualization using Apreo C Electron Microscope (Thermo Scientific, Waltham, MA, USA). All reagents were purchased from Sigma Aldrich, Carlsbad, CA, USA unless mentioned otherwise.

### 4.8. Immunostaining and Confocal Microscopy

Fibroblasts, iMSCs, and PDGFRα-positive cells were grown on sterile cover slips and fixed in 4% paraformaldehyde (Sigma Aldrich, St. Louis, MO, USA) for 15 min at room temperature, following which the cells were washed three times with ice-cold PBS (Sigma Aldrich, St. Louis, MO, USA) for 5 min each. Fixed cells were blocked with 1% bovine serum albumin (VWR, Radnor, PA, USA) for 30 min to unblock any unspecific binding. Cells were then washed with PBS for 10 min and incubated with the following primary antibodies in a humidified chamber overnight at 4 °C: anti-CD34 (ab81289; Abcam, Cambridge, UK), anti-CD44 (BD Pharmingen, San Diego, CA, USA), anti-KCa2.3-ATTO-594 (SK3; Alomone Labs, Jerusalem, Israel), anti-PDGFRα (ab234965; Abcam, Cambridge, UK), and anti-nNOS (3G6B10; Invitrogen Carlsbad, CA, USA). Post incubation, cells were washed three times in PBST for 10 min following which the cells were incubated with appropriate secondary antibodies: Goat anti-rabbit IgG H&L (Alexa Fluorfi 488; ab150077; Abcam, Cambridge, UK) and goat anti-mouse IgG H&L (Alexa Fluorfi 594; ab150116; Abcam, Cambridge, UK) for 1 h in dark at room temperature. The secondary antibodies solution was discarded, and the cells were washed three times using PBST to remove any excess binding. Cells were then mounted on the slides using Slow Fade™ Gold Antifade mountant (Thermo Fisher Scientific, Waltham, MA, USA) and screened on a Nikon Eclipse TI-A scanning confocal Microscope (Nikon, Tokyo, Japan) using a 60× oil immersion lens. Images were obtained using NIS elements microscope imaging software (Nikon, Tokyo, Japan). The images were analyzed using ImageJ. The acquisition of the images was in 2D and the cells were seeded on coverslips and we used 2D, which reflects the aim of the study and it is the most widely used presentation in most of the previous studies [59]. We acquired almost 30 cells per treatment, using the same exact exposure time for the used lasers between the different samples and different fields within the same sample. The lasers excitation emission for our targets were DAPI (425/475 nm), Alexa Fluorfi 488 (500/550 nm), and Alexa Fluorfi 594 (590/617).

### 4.9. Statistical Analysis

All data are expressed as mean ± standard error and differences between individual means were assessed using one-way analysis of variance (ANOVA) followed by the Tukey Multiple Comparisons tests. Probability values of *p* < 0.05 were considered as statistically significant.

### 4.10. Limitations

The conduction of further experiments to assess the outcomes of some of immunostaining and confocal microscopy was limited due the outbreak of Coronavirus pandemic, which led to the cancellation of several shipments of reagents and consumables. In addition, the access to the lab facilities was limited to maintain the required capacity.

## Figures and Tables

**Table 1:** Primers used for determining fibroblastic differentiation using quantitative real-time PCR.

Gene	Forward Primer (5′–3′)	Reverse Primer (5′–3′)	GenBank No.	Product Size (bp)
COL I	AACAAATAAGCCATCACGCCT	TGAAACAGACTGGGCCAATGTC	NM_000089	101
ELA	AAAGCAGCAGCAAAGTTCGG	ACCTGGGAC AACTGGAATCC	NM_001081755	274
DEC	GATGCAGCTAGC CTGAAAGG	TCACACCCGAATAAGAAGCC	NM_133503	274
TIMP1	TTTCTTGGTTCCCCAGAATG	CAGAGCTGCAGAGCAACAAG	NG_012533	99
HAS3	TGTGCAGTGTATTAGTGGGCCCTT	TTGGAGCGCGTATACTTAGTT	NM_005329	177
CD44	TGCCGCTTTGCAGGTGTAT	GGCCTCCGTCCGAGAGA	NM_001001392	70
FSP-1	AGCTTCTTGGGGAAAAGGAC	CCCCAACCACAT CAGAGG	NM_019554	200
ALP	TGGAGCTTCAGAAGCTCAACACCA	ATCTCGTTGTCTGAGTACCAGTCC	NM_000478	454
AGG	TCGAGGACAGCGAGGCC	TCGAGGGTGTAGCGTGTAGAGA	NM_013227	85
p53	TGCGTGTGGAGTATTTGGATG	GTGTGATGATGGTGAGGATGG	NM_000546	168
GAPDH	GAAATCCCATCACCATCTTCCAGG	GAGCCCCAGCCTTCTCCATG	NM_002046	120

## Data Availability

The data presented in this study are available on request from the corresponding author.

## Abbreviations

AGG	Aggrecan
ALP	Alkaline Phosphatase
COL I	Collagen type I.
CTGF	Connective Tissue Growth Factor
DEC	Decorin.
DM	Diabetes Mellitus
ECM	Extracellular Matrix
EDTA	Ethylenediaminetetraacetic acid
ELA	Elastin.
PDGFRα-positive cells	Platelet-derived growth factor receptor-α-positive cells
FSP-1	Fibroblast Specific Protein-1
HAS3	Hyaluronic Acid Synthase 3
iMSCs	Telomerase transformed mesenchymal stromal cells
LAA	L-ascorbic acids
LNNA	L-NG-Nitro-L-Arginine
iMSCs	telomerase transformed mesenchymal stromal cells
NO	Nitric Oxide
nNOS	Neuronal Nitric Oxide Synthase
PDGFRα	Platelet-Derived Growth Factor Receptor alpha
SCD	Stem Cell Differentiation
SK3	Small conductance calcium-activated potassium channel
SNAP	S-nitroso-N-Acetylpenicillamine
TIMP1	Tissue Inhibitor of Metalloproteinases 1

## References

[1] Camilleri M. (2007). Diabetic Gastroparesis. N. Engl. J. Med..

[2] Parkman H.P., Fass R., Foxx-Orenstein A.E. (2010). Treatment of Patients with Diabetic Gastroparesis. Gastroenterol. Hepatol..

[3] Homko C., Siraj E.S., Parkman H.P. (2016). The impact of gastroparesis on diabetes control: Patient perceptions. J. Diabetes Complicat..

[4] Talley N.J., Young L., Bytzer P., Bytzer P., Hammer J., Leemon M., Jones M., Horowitz M. (2001). Impact of chronic gastrointestinal symptoms in diabetes mellitus on health-related quality of life. Am. J. Gastroenterol..

[5] Mussa B.M., Sood S., Verberne A.J. (2018). Implication of neurohormonal-coupled mechanisms of gastric emptying and pancre-atic secretory function in diabetic gastroparesis. World J. Gastroenterol..

[6] Sanders K.M. (1996). A case for interstitial cells of Cajal as pacemakers and mediators of neurotransmission in the gastrointestinal tract. Gastroenterology.

[7] Vittal H., Farrugia G., Gomez G., Pasricha P.J. (2007). Mechanisms of Disease: The pathological basis of gastroparesis—A review of experimental and clinical studies. Nat. Clin. Pr. Gastroenterol. Hepatol..

[8] Sanders K.M., Koh S.D., Ward S.M. (2006). Interstitial Cells of Cajal as Pacemakers in the Gastrointestinal Tract. Annu. Rev. Physiol..

[9] Vannucchi M.G. (2020). The Telocytes: Ten Years after Their Introduction in the Scientific Literature. An Update on Their Morphology, Distribution, and Potential Roles in the Gut. Int. J. Mol. Sci..

[10] Horiguchi K., Komuro T. (2000). Ultrastructural observations of fibroblast-like cells forming gap junctions in the W/W(nu) mouse small intestine. J. Auton. Nerv. Syst..

[11] Iino S., Horiguchi K., Horiguchi S., Nojyo Y. (2009). c-Kit-negative PDGFRα-positive express platelet-derived growth factor receptor α in the murine gastrointestinal musculature. Histochem. Cell Biol..

[12] Iino S., Nojyo Y. (2009). Immunohistochemical demonstration of c-Kit-negative PDGFRα-positive in murine gastrointestinal musculature. Arch Histol. Cytol..

[13] Kurahashi M., Zheng H., Dwyer L., Ward S.M., Koh S.D., Sanders K.M. (2011). A functional role for the ‘fibroblast-like cells’ in gastrointestinal smooth muscles. J. Physiol..

[14] Grover M., Bernard C.E., Pasricha P.J., Parkman H.P., Abell T.L., Nguyen L.A., Snape W., Shen K.R., Sarr M., Swain J. (2012). Platelet-derived growth factor receptor α (PDGFRα)-expressing “fibro-blast-like cells” in diabetic and idiopathic gastroparesis of humans. J. Neurogastroenterol. Motil..

[15] Gevaert T., Vanstreels E., Daelemans D., Franken J., Van Der Aa F., Roskams T., De Ridder D. (2014). Identification of Different Phenotypes of Interstitial Cells in the Upper and Deep Lamina Propria of the Human Bladder Dome. J. Urol..

[16] Lu C., Huang X., Lu H.L., Liu S.-H., Zang J.-Y., Li Y.-J., Chen J., Xu W.-X. (2018). Different distributions of interstitial cells of Cajal and platelet-derived growth factor receptor-α positive cells in colonic smooth muscle cell/interstitial cell of Cajal/platelet-derived growth factor receptor-α positive cell syncytium in mice. World J. Gastroenterol..

[17] Kurahashi M., Nakano Y., Hennig G.W., Ward S.W., Sanders K.M. (2012). Platelet-derived growth factor receptor α-positive cells in the tunica mus-cularis of human colon. J. Cell Mol. Med..

[18] Blair P.J., Rhee P.-L., Sanders K.M., Ward A.S.M. (2014). The Significance of Interstitial Cells in Neurogastroenterology. J. Neurogastroenterol. Motil..

[19] Groneberg D., Voussen B., Friebe A. (2016). Integrative Control of Gastrointestinal Motility by Nitric Oxide. Curr. Med. Chem..

[20] Groneberg D., König P., Koesling D., Friebe A. (2011). Nitric Oxide–Sensitive Guanylyl Cyclase Is Dispensable for Nitrergic Signaling and Gut Motility in Mouse Intestinal Smooth Muscle. Gastroenterology.

[21] Mussa B.M., Sartor D.M., Rantzau C., Verberne A.J. (2011). Effects of nitric oxide synthase blockade on dorsal vagal stimulation-induced pancreatic insulin secretion. Brain Res..

[22] Watkins C.C., Sawa A., Jaffrey S., Blackshaw S., Barrow R.K., Snyder S.H., Ferris C.D. (2000). Insulin restores neuronal nitric oxide synthase expression and function that is lost in diabetic gastropathy. J. Clin. Investig..

[23] Choi K.M., Gibbons S.J., Roeder J.L., Lurken M.S., Zhu J., Wouters M.M., Miller S.M., Szurszewski J.H., Farrugia G. (2007). Regulation of interstitial cells of Cajal in the mouse gastric body by neuronal nitric oxide. Neurogastroenterol. Motil..

[24] Choi K.M., Gibbons S.J., Nguyen T.V., Stoltz G.J., Lurken M.S., Ordog T., Szurszewski J.H., Farrugia G. (2008). Heme Oxygenase-1 Protects Interstitial Cells of Cajal From Oxidative Stress and Reverses Diabetic Gastroparesis. Gastroenterology.

[25] Tong Z., Sant S., Khademhosseini A., Jia X. (2011). Controlling the Fibroblastic Differentiation of Mesenchymal Stem Cells Via the Combination of Fibrous Scaffolds and Connective Tissue Growth Factor. Tissue Eng. Part A.

[26] Xu C., Jiang J., Sottile V., McWhir J., Lebkowski J., Carpenter M.K. (2004). Immortalized Fibroblast-Like Cells Derived from Human Embryonic Stem Cells Support Undifferentiated Cell Growth. Stem Cells.

[27] Wang Q., Zhang W., He G., Sha H., Quan Z. (2016). Method for in vitro differentiation of bone marrow mesenchymal stem cells into endothe-lial progenitor cells and vascular endothelial cells. Mol. Med. Rep..

[28] Ozen A., Sancak I.G., Tiryaki M., Ceylan A., Pinarli F.A., Delibasi T. (2014). Mesenchymal Stem Cells (Mscs) in Scanning Electron Microscopy (SEM) World. Niche.

[29] Denu R.A., Nemcek S., Bloom D.D., Goodrich A.D., Kim D.F., Hematti P. (2016). Fibroblasts and Mesenchymal Stromal/Stem Cells Are Phenotypically Indistinguishable. Acta Haematol..

[30] Ichim T.E., O’Heeron P., Kesari S. (2018). Fibroblasts as a practical alternative to mesenchymal stem cells. J. Transl. Med..

[31] Yoon D., Sim H., Hwag I., Lee J.-S., Chun W. (2018). Accelerated Wound Healing by Fibroblasts Differentiated from Human Embryonic Stem Cell-Derived Mesenchymal Stem Cells in a Pressure Ulcer Animal Model. Stem Cells Int..

[32] Heng E.C., Huang Y., Black S.A., Trackman P.C. (2006). CCN2, connective tissue growth factor, stimulates collagen deposition by gingival fibroblasts via module 3 and alpha6- and beta1 integrins. J. Cell Biochem..

[33] Tajima S., Izumi T. (1996). Differential in vitro responses of elastin expression to basic fibroblast growth factor and transforming growth factor beta 1 in upper, middle and lower dermal fibroblasts. Arch. Dermatol. Res..

[34] Droguett R., Cabello-Verrugio C., Riquelme C., Brandan E. (2006). Extracellular proteoglycans modify TGF-β bio-availability attenuating its signaling during skeletal muscle differentiation. Matrix Biol..

[35] Vial C., Gutiérrez J., Santander C., Cabrera D., Brandan E. (2011). Decorin Interacts with Connective Tissue Growth Factor (CTGF)/CCN2 by LRR12 Inhibiting Its Biological Activity. J. Biol. Chem..

[36] Rilla K., Pasonen-Seppänen S., Deen A.J., Koistinen V.V., Wojciechowski S., Oikari S., Kärnä R., Bart G., Törrönen K., Tammi R.H. (2013). Hyaluronan production enhances shedding of plasma membrane-derived microvesicles. Exp. Cell Res..

[37] Yang M., Huang H., Li J., Huang W., Wang H. (2007). Connective tissue growth factor increases matrix metalloproteinase-2 and suppresses tissue inhibitor of matrix metalloproteinase-2 production by cultured renal interstitial fibroblasts. Wound Repair Regen..

[38] Yamamoto K., Kishida T., Sato Y., Nishioka K., Ejima A., Fujiwara H., Kubo T., Yamamoto T., Kanamura N., Mazda O. (2015). Direct conversion of human fibroblasts into functional osteoblasts by defined factors. Proc. Natl. Acad. Sci. USA.

[39] Qian H., Le Blanc K., Sigvardsson M. (2012). Primary Mesenchymal Stem and Progenitor Cells from Bone Marrow Lack Expression of CD44 Protein. J. Biol. Chem..

[40] Iwano M., Plieth D., Danoff T.M., Xue C., Okada H., Nelison E.C. (2002). Evidence that fibroblasts derive from epithelium during tissue fibrosis. J. Clin. Investig..

[41] Zhou D., Zhang Z., He L.-M., Du J., Zhang F., Sun C.-K., Zhou Y., Wang X.-W., Lin G., Song K.-M. (2014). Conversion of fibroblasts to neural cells by p53 depletion. Cell Rep..

[42] Houlihan D.D., Mabuchi Y., Morikawa S., Niibe K., Araki D., Suzuki S., Okano H., Matsuzaki Y. (2012). Isolation of mouse mesenchymal stem cells on the basis of expression of Sca-1 and PDGFR-α. Nat. Protoc..

[43] Lin C.-S., Ning H., Lin G., Lue T.F. (2012). Is CD34 truly a negative marker for mesenchymal stromal cells?. Cytotherapy.

[44] Scherberich A., Di Di Maggio N., McNagny K.M. (2013). A familiar stranger: CD34 expression and putative functions in SVF cells of adipose tissue. World J. Stem Cells.

[45] Liebau S., Tischendorf M., Ansorge D., Linta L., Stockmann M., Weidgang C., Iacovino M., Boeckers T., Von Wichert G., Kyba M. (2011). An Inducible Expression System of the Calcium-Activated Potassium Channel 4 to Study the Differential Impact on Embryonic Stem Cells. Stem Cells Int..

[46] Pchelintseva E., Djamgoz M.B.M.B.A. (2018). Mesenchymal stem cell differentiation: Control by calcium-activated potassium channels. J. Cell. Physiol..

[47] Iino S., Horiguchi K., Nojyo Y. (2008). Interstitial cells of Cajal are innervated by nitrergic nerves and express nitric ox-ide-sensitive guanylate cyclase in the guinea-pig gastrointestinal tract. Neuroscience.

[48] Kwesiga M.P., Cook E., Hannon J., Wayward S., Gwaltney C., Rao S., Frost M.C. (2018). Investigative Study on Nitric Oxide Production in Human Dermal Fibroblast Cells under Normal and High Glucose Conditions. Med. Sci..

[49] Mu K., Yu S., Kitts D.D. (2019). The Role of Nitric Oxide in Regulating Intestinal Redox Status and Intestinal Epithelial Cell Functionality. Int. J. Mol. Sci..

[50] Murphy M.P. (1999). Nitric oxide and cell death. Biochim. Biophys. Acta (BBA)-Bioenerg..

[51] Gross S.S., Wolin M.S. (1995). Nitric oxide: Pathophysiological mechanisms. Ann. Rev. Physiol..

[52] Kröncke K.-D., Fehsel K., Kolb-Bachofen V. (1997). Nitric Oxide: Cytotoxicity versus Cytoprotection—How, Why, When, and Where?. Nitric Oxide.

[53] Iadecola C. (1997). Bright and dark sides of nitric oxide in ischemic brain injury. Trends Neurosci..

[54] Stark M.E., Szurszewski J.H. (1992). Role of nitric oxide in gastrointestinal and hepatic function and disease. Gastroenterology.

[55] Trento C., Marigo I., Pievani A., Galleu A., Dolcetti L., Wang C.-Y., Serafini M., Bronte V., Dazzi F. (2017). Bone marrow mesenchymal stromal cells induce nitric oxide synthase-dependent differentiation of CD11b+ cells that expedite hematopoietic recovery. Haematologica.

[56] Chen L., Zhang Y., Tao L., Yang Z., Wang L. (2016). Mesenchymal Stem Cells with eNOS Over-Expression Enhance Cardiac Repair in Rats with Myocardial Infarction. Cardiovasc. Drugs Ther..

[57] Pacher P., Beckman J.S., Liaudet L. (2007). Nitric Oxide and Peroxynitrite in Health and Disease. Physiol. Rev..

[58] Adela R., Nethi S.K., Bagul P.K., Barui A.K., Mattapally S., Kuncha M., Patra C.R., Reddy P.N.C., Banerjee S.K. (2015). Hyperglycaemia Enhances Nitric Oxide Production in Diabetes: A Study from South Indian Patients. PLoS ONE.

[59] Hameed M., Panicker S., Abdallah S.H., Khan A.A., Han C., Chehimi M.M., Mohamed A.A. (2020). Protein-Coated Aryl Modified Gold Nanoparticles for Cellular Uptake Study by Osteosarcoma Cancer Cells. Langmuir.

